# Supplementary figures and images for: Prevalence of Salmonella spp. and Escherichia coli in the feces of free-roaming wildlife throughout South Korea
Source: PLoS One. 2024 Feb 15;19(2):e0281006. doi: 10.1371/journal.pone.0281006 (PMC10868816; doi:10.1371/journal.pone.0281006)

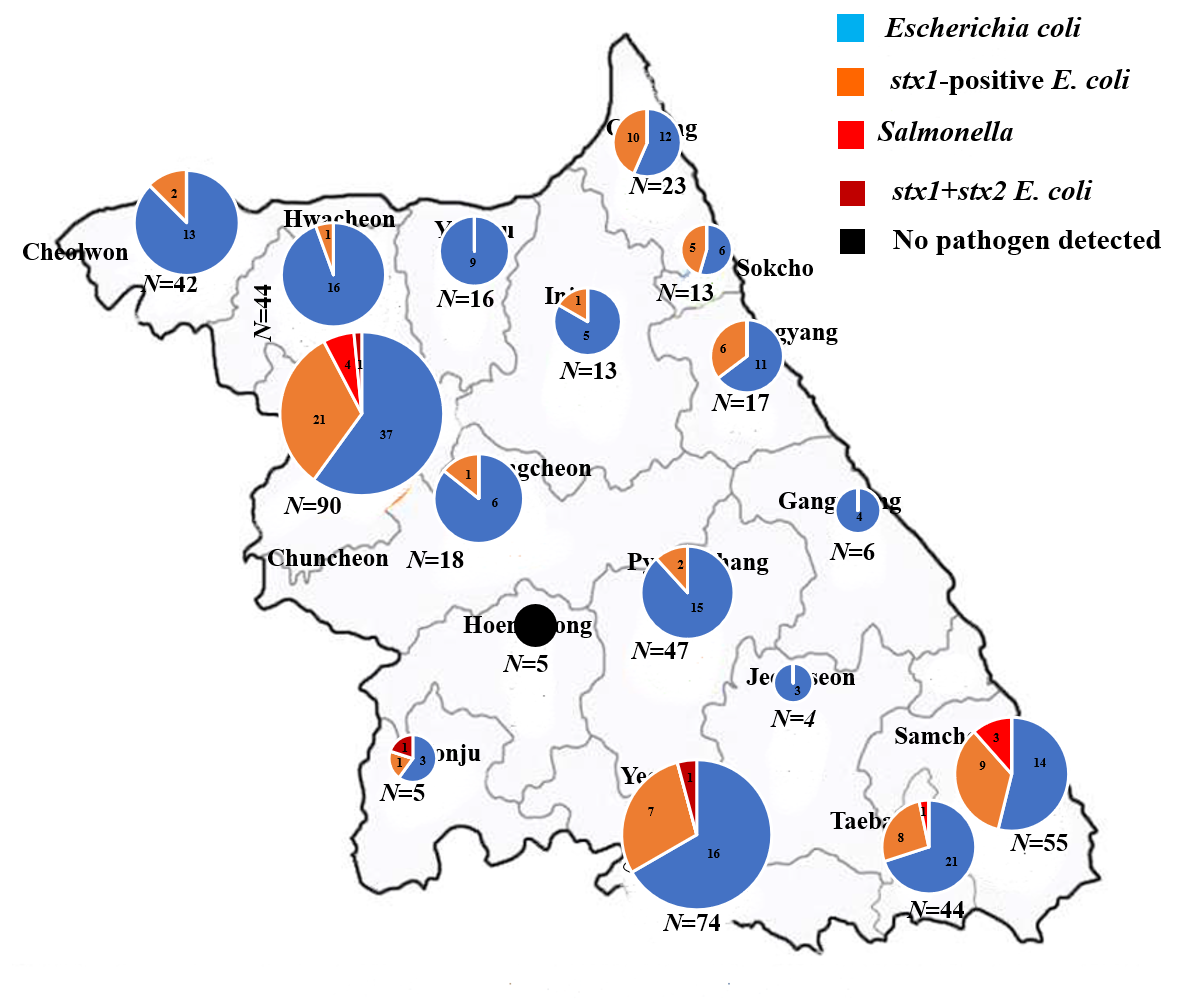

Supplement: S1 Fig — “N” = A total collection samples in each region and color-coded round shape ratio indicate Escherichia coli (blue), stx1-detected E. coli (orange), stx1 and stx2-detected E. coli (red-orange), Salmonella (red), respectively. (TIF) [file pone.0281006.s001.tif]

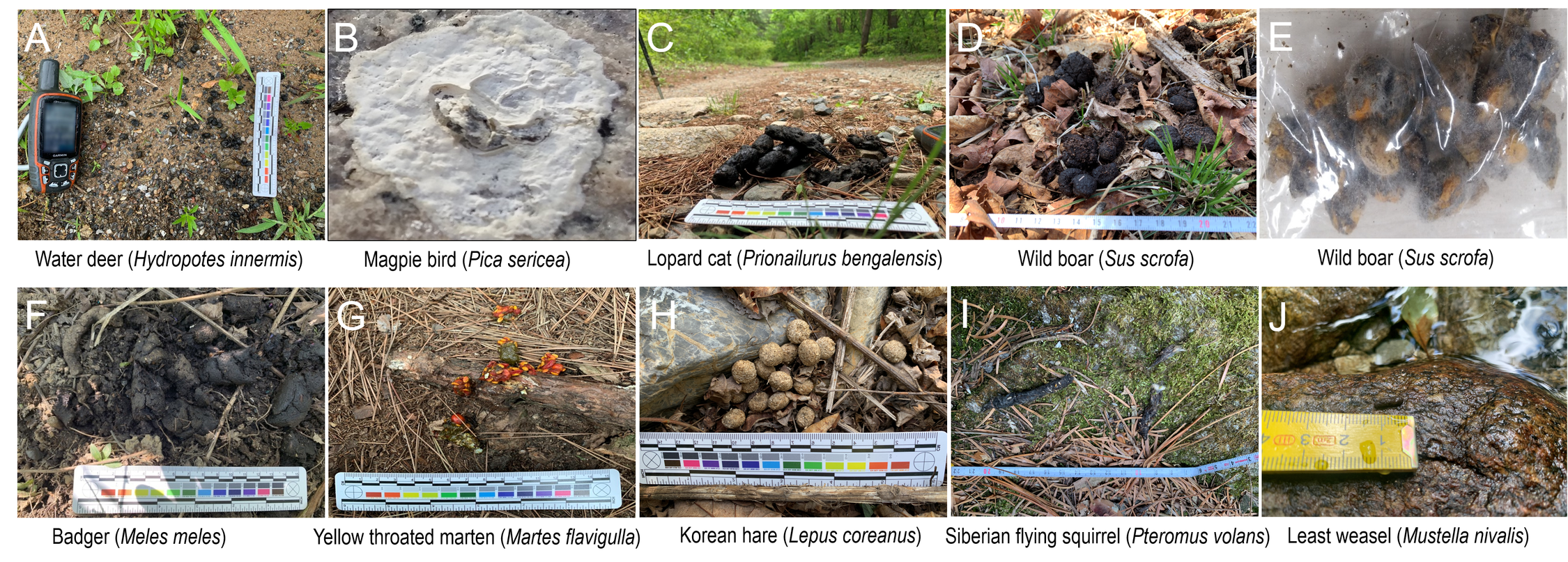

Supplement: S2 Fig — Shows the normal structure and color of collected feces samples A) water dear (H. inermis) feces in a round shape or heart shape with convex wall B) white urea-containing liquid with an amorphous shape of Pica sericea feces C) leopard cat fecal tubular shape of leopard cat (Prionailurus bengalensis) D) wild boar (Sus scrofa) feces E). wild boar (Sus scrofa) fecal with corn seed F) badger (Meles meles) feces with tubular-shape G) yellow throated marten (Martes flavigulla) H) Korean hare (Lepus coreanus) I). flying squirrel (Pteromus volans) and J). least weasel (Mustella nivalis). (TIF) [file pone.0281006.s002.tif]

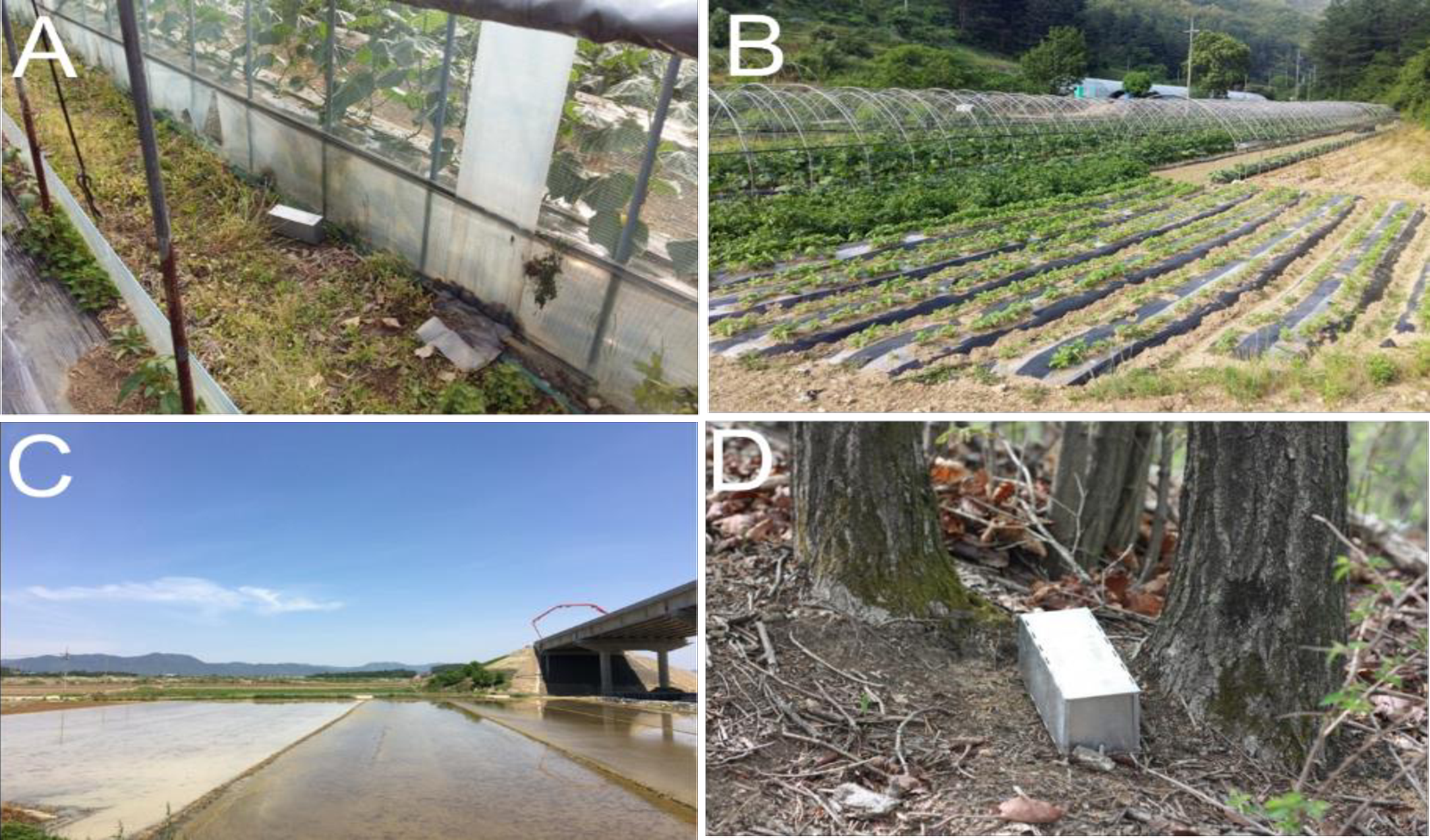

Supplement: S3 Fig — A–D. The sample collection sites, A) Sherman traps were kept in an orchard garden, Gangwon-do, B) Survey area of the Chuncheon agricultural plots, Gangwon-do, C) Survey area of the nearby streams of water where samples are collected, and D) Survey area of Bukhansan, a forested region where samples are collected. (TIF) [file pone.0281006.s003.tif]

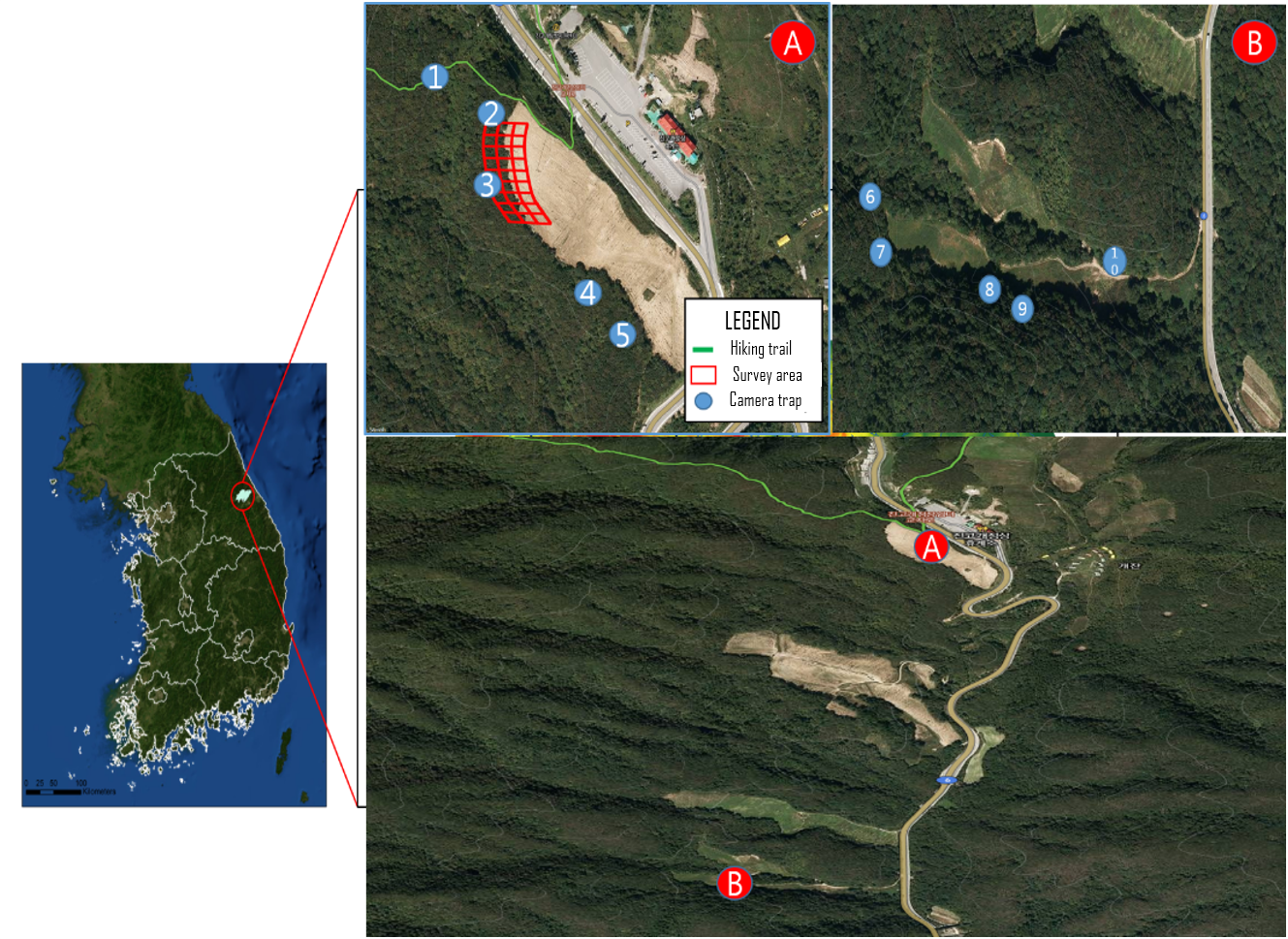

Supplement: S4 Fig — 1–10 represent camera trapping, and A and B indicate survey area location. (TIF) [file pone.0281006.s004.tif]

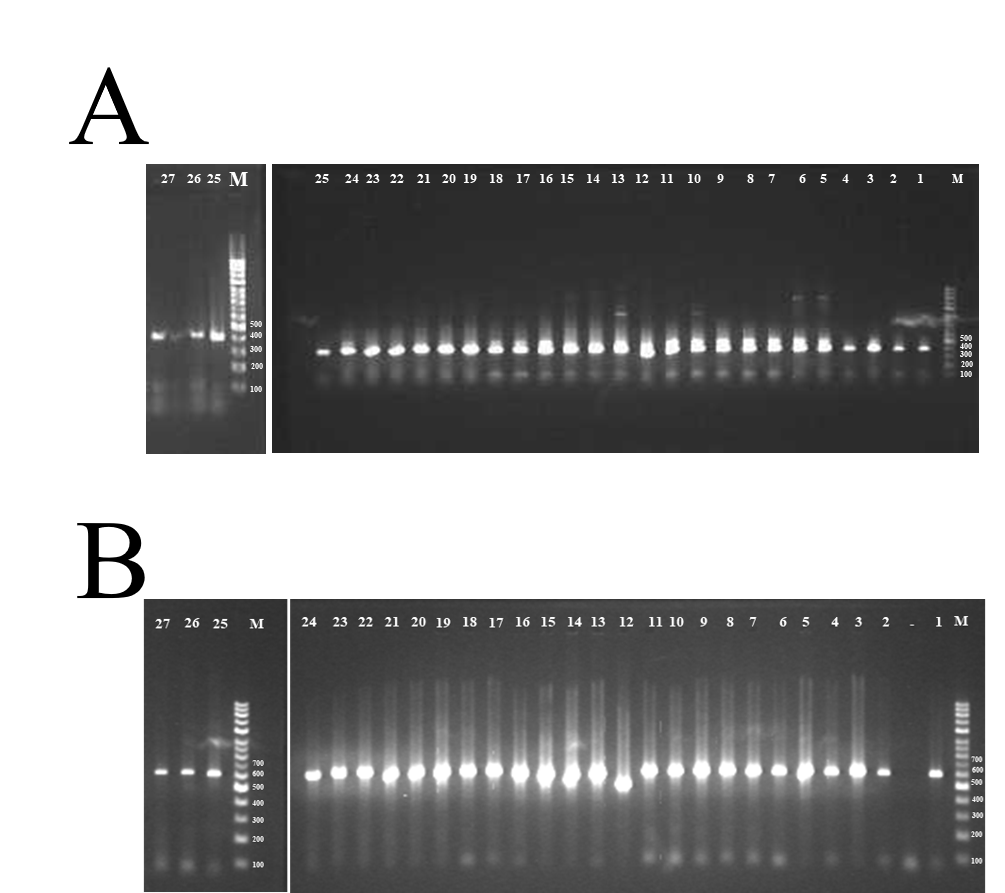

Supplement: S5 Fig — (A, B). PCR amplification for Salmonella detection with invasion gene (invA) primer set PCR bands were observed in all 27 single colonies. The 27 isolated strains (n = 21, the isolated strains from wild animal fecal sources and the most widely prevalent reference Salmonella serovars, n = 6) were used. PCR band ‘M’ indicates DNA 100 bp marker. The target band of the amplified invA primer was 398 bp. The gel lane numbers are as follows: from No.1 to 6 reference serovars and Salmonella-positive isolated strains were detected from wild animal fecal between lanes No.7 and No.27 (B) PCR amplification for Salmonella detection with the iron chelating (iroB) gene primer set. PCR bands were observed in all 27 single colonies. PCR band ‘M’ indicates DNA 100 bp marker. The target band of the amplified iroB primer was 606 bp. The Lane from No.1 to 6 reference serovars and Salmonella-positive isolated strains were detected from wild animal fecal between lanes from No.7 and No.27. The symbol “–“PCR mixture without genomic DNA of samples was used as a negative control. Each sample is indicated by a host individual ID with bacterial colony ID in parentheses. Lane from No.1 to 6 reference serovars [S. Abony (BA1800061), S. enteritidis (NCCP-14545), S. Agona (NCCP-12231), S. Typhimurium (NCCP-14760), S. Typhi (NCCP-14641), and S. Enterica (NCCP-15756)]; (Lane No. 7 = CaPrBe_3 (WC_A3); Lane No. 8 = CaPrBe_5 (WC-A1-5-1); Lane No. 9 = CaPrBe_10 (WC-A1-5-P); Lane No. 10 = CaPrBe_19 (WC-B4-5-2); Lane No. 11 = CaPrBe_26 (WC-B4-8-2); Lane No. 12 = CaPrBe_27 (WCB4_4); Lane No. 13 = CaPrBe_28 (WC-B4-5-1); Lane No. 14 = CaPrBe_31 (WC-B4-8-1); Lane No. 15 = CaPrBe_39 (WC_B4(16); Lane No. 16 = SuSuSc_1 (WB_235); Lane No. 17 = SuSuSc_2 (WB_241); Lane No. 18 = CaMeMe_1 (Badger-derong-I); Lane No. 19 = CaMeMe_3 (Raccon-BD-VI); Lane No. 20 = CaMeMe_4 (Raccon/BD-I); Lane No. 21 = CaMeMe_9 (badger-derong-100); Lane No. 22 = ABF_42 (BF-3-KNU-I); Lane No. 23 = ABF_43 (BF_3_KNU(ii)); Lane No. 24 = ABF_47 [file pone.0281006.s005.tif]

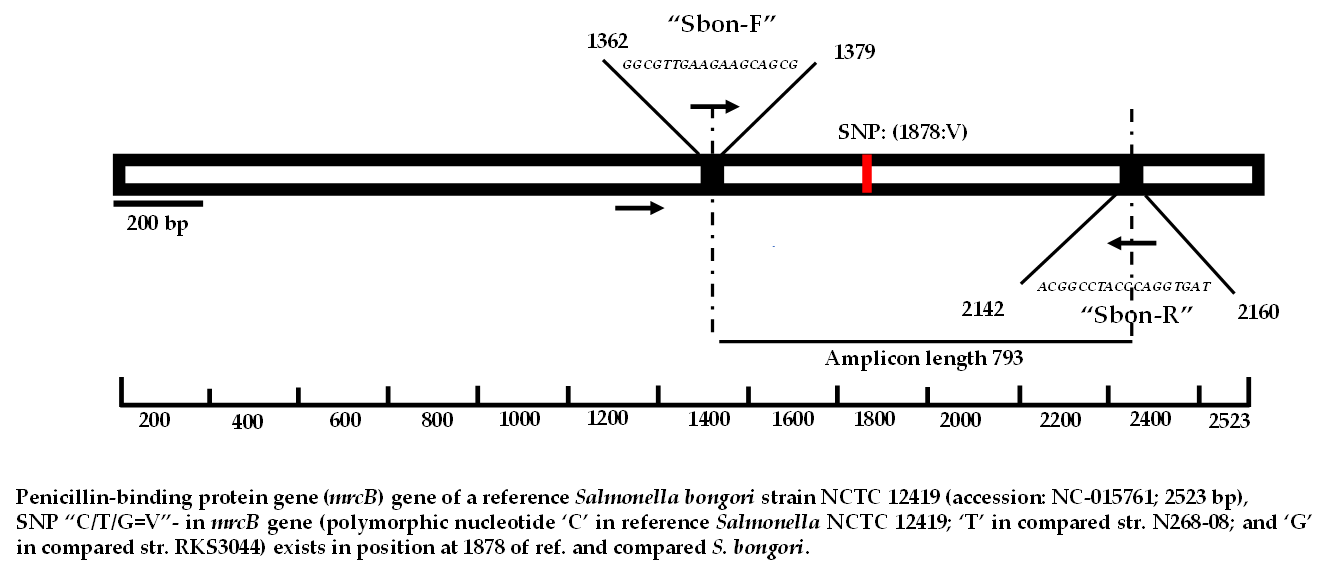

Supplement: S6 Fig — The first primer set of forward primer, Sbon-F (19 bp) was between 1362 and 1379, and reverse primer Sbon-R (21 bp) was between 2160 and 2142, respectively and the target band was approximately 793 bp. Red color “SNP-1878” indicates the bases either T/ C/G mutated/replaced with each other. The natural encompassing SNP position was marked by red color in the position of 1878 of the reference (NC-015761) mrcB gene sequence. (TIF) [file pone.0281006.s006.tif]

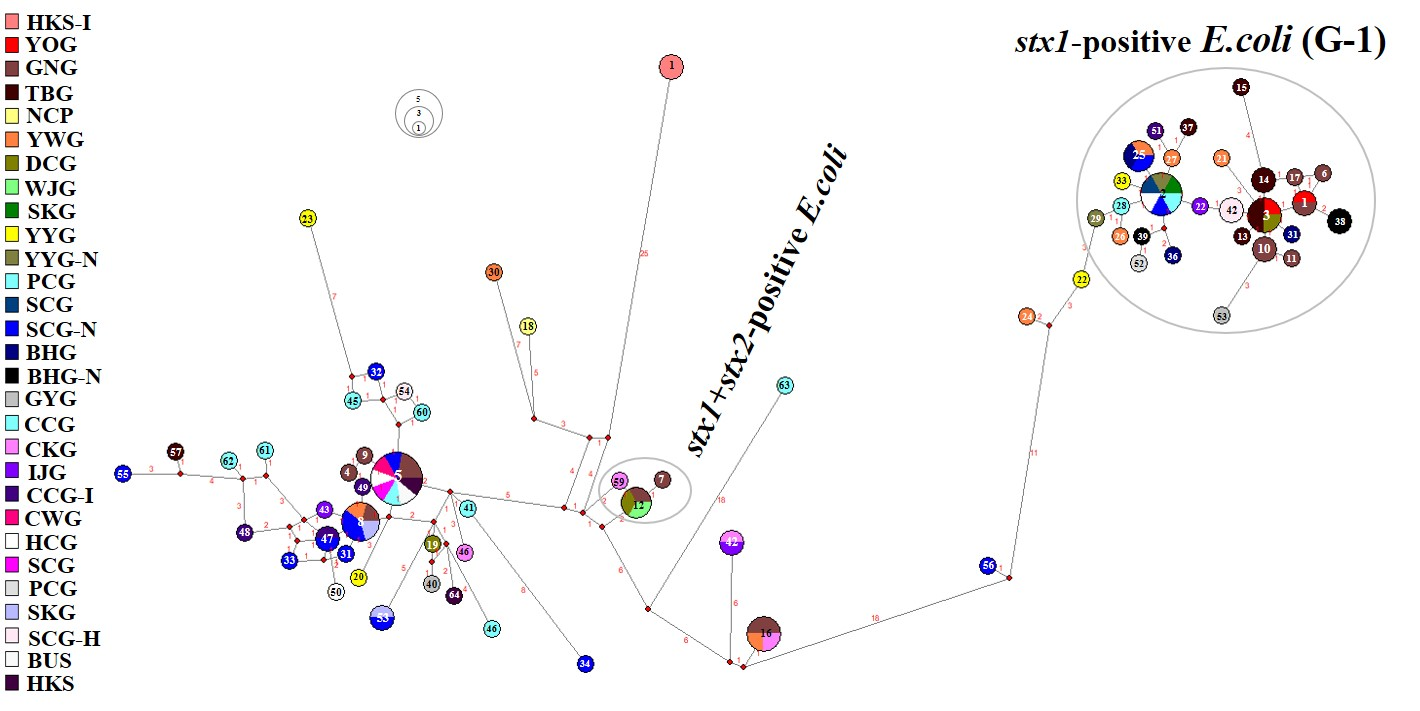

Supplement: S7 Fig — Distribution pattern (locality) of bacterial-borne zoonotic pathogen Shiga toxin genes (stx1 and stx1+stx2)-detected E. coli from the feces of wild mammals and birds in South Korea. (TIF) [file pone.0281006.s007.tif]
