# Supplementary material for: Prevalence of Salmonella spp. and Escherichia coli in the feces of free-roaming wildlife throughout South Korea
Source: PLoS One. 2024 Feb 15;19(2):e0281006. doi: 10.1371/journal.pone.0281006 (PMC10868816; doi:10.1371/journal.pone.0281006)
Supplement: S1 Table — (DOCX) [file pone.0281006.s008.docx]

**S1 Table****.** Geographic location and haplotype information of *stx-1*-detected *Escherichia coli* isolated from wildlife feces in South Korea

| Northeastern South Korea (NES) | Chuncheon, Gangwon-do | CCG | Hap-5 | 1 ^#^ |
| --- | --- | --- | --- | --- |
|  | Chuncheon, Gangwon-do | CCG | Hap-7 | 1 |
|  | Chuncheon, Gangwon-do | CCG | Hap-8 | 1 |
|  | Chuncheon, Gangwon-do | CCG | Hap-9 | 1 |
|  | Chuncheon, Gangwon-do | CCG | Hap-41 | 1 |
|  | Chuncheon, Gangwon-do | CCG | Hap-45 | 1 |
|  | Chuncheon, Gangwon-do | CCG | Hap-46 | 1 |
|  | Chuncheon, Gangwon-do | CCG | Hap-47 | 1 |
|  | Chuncheon, Gangwon-do | CCG | Hap-48 | 1 |
|  | Chuncheon, Gangwon-do | CCG | Hap-49 | 1 |
|  | Chuncheon, Gangwon-do | CCG | Hap-51 | 1 |
|  | Chuncheon, Gangwon-do | CCG | Hap-60 | 1 |
|  | Chuncheon, Gangwon-do | CCG | Hap-61 | 1 |
|  | Chuncheon, Gangwon-do | CCG | Hap-62 | 1 |
|  | Mt. Daeryong, Chuncheon, Gangwon-do | CCG-I | Hap-2 | 1 |
|  | Mt. Daeryong, Chuncheon, Gangwon-do | CCG-I | Hap-12 | 1 |
|  | Mt. Daeryong, Chuncheon, Gangwon-do | CCG-I | Hap-19 | 1 |
|  | Mt. Daeryong, Chuncheon, Gangwon-do | CCG-I | Hap-47 | 1 |
|  | Mt. Daeryong, Chuncheon, Gangwon-do | CCG-I | Hap-48 | 1 |
|  | Mt. Daeryong, Chuncheon, Gangwon-do | CCG-I | Hap-49 | 1 |
|  | Mt. Daeryong, Chuncheon, Gangwon-do | CCG-I | Hap-51 | 1 |
|  | Goseong, Gangwon-do | GSG | Hap-2 | 1 |
|  | Goseong, Gangwon-do | GSG | Hap-5 | 1 |
|  | Goseong, Gangwon-do | GSG | Hap-8 | 2 |
|  | Goseong, Gangwon-do | GSG | Hap-25 | 1 |
|  | Goseong, Gangwon-do | GSG | Hap-31 | 1 |
|  | Goseong, Gangwon-do | GSG | Hap-32 | 1 |
|  | Goseong, Gangwon-do | GSG | Hap-33 | 1 |
|  | Goseong, Gangwon-do | GSG | Hap-34 | 1 |
|  | Goseong, Gangwon-do | GSG | Hap-55 | 1 |
|  | Goseong, Gangwon-do | GSG | Hap-56 | 1 |
|  | Chamchak, Gangwon-do | SCG | Hap-2 | 3 |
|  | Chamchak, Gangwon-do | SCG | Hap-5 | 1 |
|  | Chamchak, Gangwon-do | SCG | Hap-54 | 1 |
|  | Mt. Seorak, Sokcho, Gangwon-do | SKG | Hap-2 | 1 |
|  | Mt. Seorak, Sokcho, Gangwon-do | SKG | Hap-8 | 1 |
|  | Mt. Seorak, Sokcho, Gangwon-do | SKG | Hap-53 | 1 |
|  | Chokso, Gangwon-do | SKG-I | Hap-16 | 1 |
|  | Chokso, Gangwon-do | SKG-I | Hap-42 | 1 |
|  | Chokso, Gangwon-do | SKG-I | Hap-59 | 1 |
|  | Yangyang, Gangwon-do | YYG | Hap-3 | 1 |
|  | Yangyang, Gangwon-do | YYG | Hap-20 | 1 |
|  | Yangyang, Gangwon-do | YYG | Hap-22 | 1 |
|  | Yangyang, Gangwon-do | YYG | Hap-2 | 1 |
|  | Yangyang, Gangwon-do | YYG | Hap-29 | 1 |
|  | Yangyang, Gangwon-do | YYG | Hap-36 | 1 |
|  | Wonju, Gangwon-do | WJG | Hap-12 | 1 |
|  | Injegun, Gangwon-do | IJG | Hap-42 | 1 |
|  | Injegun, Gangwon-do | IJG | Hap-43 | 1 |
|  | Pyeongchang, Gangwon-do | PCG | Hap-28 | 1 |
|  | Pyeongchang, Gangwon-do | PCG | Hap-63 | 1 |
|  | Hwacheon, Gangwon-do | HCG | Hap-2 | 1 |
|  | Hongcheon, Gangwon-do | HNG | Hap-5 | 1 |
| Middleeastern South Korea (MES) | Taebeksan, Gangwon-do | TBG | Hap-2 | 1 |
|  | Taebeksan, Gangwon-do | TBG | Hap-13 | 1 |
|  | Taebeksan, Gangwon-do | TBG | Hap-14 | 2 |
|  | Taebeksan, Gangwon-do | TBG | Hap-15 | 1 |
|  | Taebeksan, Gangwon-do | TBG | Hap-37 | 1 |
|  | Taebeksan, Gangwon-do | TBG | Hap-57 | 1 |
|  | Youngwol, Gangwon-do | YWG | Hap-8 | 1 |
|  | Youngwol, Gangwon-do | YWG | Hap-16 | 1 |
|  | Youngwol, Gangwon-do | YWG | Hap-21 | 1 |
|  | Youngwol, Gangwon-do | YWG | Hap-23 | 1 |
|  | Youngwol, Gangwon-do | YWG | Hap-24 | 1 |
|  | Youngwol, Gangwon-do | YWG | Hap-25 | 1 |
|  | Youngwol, Gangwon-do | YWG | Hap-26 | 1 |
|  | Youngwol, Gangwon-do | YWG | Hap-27 | 1 |
|  | Youngwol, Gangwon-do | YWG | Hap-30 | 1 |
| Southeastern South Korea (SES) | Geochang, Gyeongsangnamdo | GNG | Hap-4 | 1 |
|  | Geochang, Gyeongsangnamdo | GNG | Hap-5 | 2 |
|  | Geochang, Gyeongsangnamdo | GNG | Hap-6 | 1 |
|  | Geochang, Gyeongsangnamdo | GNG | Hap-7 | 1 |
|  | Geochang, Gyeongsangnamdo | GNG | Hap-8 | 1 |
|  | Geochang, Gyeongsangnamdo | GNG | Hap-9 | 1 |
|  | Geochang, Gyeongsangnamdo | GNG | Hap-10 | 1 |
|  | Geochang, Gyeongsangnamdo | GNG | Hap-11 | 2 |
|  | Geochang, Gyeongsangnamdo | GNG | Hap-12 | 1 |
|  | Geochang, Gyeongsangnamdo | GNG | Hap-17 | 1 |
|  | Busan city | BUS | Hap-5 | 1 |
|  | Mt. Jiri, Gure | GYG | Hap-40 | 1 |
|  | Mt. Jiri, Gure | GYG | Hap-58 | 1 |
| Northwestern South Korea (NWS) | Mt. Buckhan, Seoul | HKS-I | Hap-1 | 2 |
|  | Mt. Buckhan, Seoul | HKS-I | Hap-5 | 1 |
|  | Hanam-si-Seoul | HKS | Hap-5 | 1 |
|  | Hanam-si-Seoul | HKS | Hap-64 | 1 |
|  | Cheolwon,Gangwon-do | CWG | Hap-5 | 1 |
| Central South Korea (CTS) | Sobaeksan, Yeongju, Gyeongsangbuk-do | YOG | Hap-2 | 1 |
|  | Sobaeksan, Yeongju, Gyeongsangbuk-do | YOG | Hap-3 | 1 |
|  | Bonghwa, Gyeongsang-buk-do | BHG | Hap-25 | 1 |
|  | Bonghwa, Gyeongsang-buk-do | BHG | Hap-39 | 1 |
|  | Jinan, Jeollabuk-do | JLD | Hap-18 | 1 |

# *stx-1* detected 93 samples isolated at least one sequence, but some isolates had multiple sequences.
