# Supplementary material for: Prevalence of Salmonella spp. and Escherichia coli in the feces of free-roaming wildlife throughout South Korea
Source: PLoS One. 2024 Feb 15;19(2):e0281006. doi: 10.1371/journal.pone.0281006 (PMC10868816; doi:10.1371/journal.pone.0281006)
Supplement: S2 Table — (DOCX) [file pone.0281006.s009.docx]

**S2 Table**. Information on Shiga toxin genes (*stx1*), and (*stx1*+*stx2*)-detected *Escherichia coli* positive samples

| No. Indiv. | English name | Scientific Name | Fecal ID | Traditional Detection^#^ | | | PCR band detection^$^ | | |  | Collection Region |
| --- | --- | --- | --- | --- | --- | --- | --- | --- | --- | --- | --- |
|  |  |  |  | Culture | | Biochemical | Universal marker | Shiga toxin-producing *E. coli* | | GenBank (Accession No.)^@^ |  |
|  |  |  |  | EMB | CT-SMAC | TSI | 16S rRNA gene | *stx-1* | *stx-1+stx-2* |  |  |
| 1 | Striped field mouse | *A.agrarius* | MuApAg-7 | **o** | **o** | **o** | **o** | **o** | **x** | ON205867 | Geosang, Gyeongsangnam-do |
| 2 | Striped field mouse | *A.agrarius* | MuApAg-12 | **o** | **o** | **o** | **o** | **o** | **o** | ON205868 | Geosang, Gyeongsangnam-do |
| 3 | Striped field mouse | *A.agrarius* | MuApAg-13 | **o** | **o** | **o** | **o** | **o** | **x** | ON205869 | Geosang, Gyeongsangnam-do |
| 4 | Striped field mouse | *A.agrarius* | MuApAg-18 | **o** | **o** | **o** | **o** | **o** | **x** | ON205880 | Bukhansan, Seoul, Kyunggi-do |
| 5 | Striped field mouse | *A.agrarius* | MuApAg-21 | **o** | **o** | **o** | **o** | **o** | **x** | ON205852 | Yeongju, Gyeongsangnam-do |
| 6 | Striped field mouse | *A.agrarius* | MuApAg-27 | **o** | **o** | **o** | **o** | **o** | **x** | ON205865 | Yeongju, Gyeongsangnam-do |
| 7 | Wood mouse | *A. sylvaticus* | MuApSy-1 | **o** | **o** | **o** | **o** | **o** | **x** | ON205881 | Yeongju, Gyeongsangnam-do |
| 8 | Wood mouse | *A. sylvaticus* | MuApSy-2 | **o** | **o** | **o** | **o** | **o** | **x** | ON205885 | Jinan, Jeollabuk-do |
| 9 | Wood mouse | *A. sylvaticus* | MuApSy-3 | **o** | **o** | **o** | **o** | **o** | **o** | ON205879 | Geosang, Gyeongsangnam-do |
| 10 | Wood mouse | *A. sylvaticus* | MuApSy-4 | **o** | **o** | **o** | **o** | **o** | **x** | ON205917 | Taebaeksan, Gangwon-do |
| 11 | Brown rat | *R. norvegicus* | MuRaNo-1 | **o** | **o** | **o** | **o** | **o** | **x** | ON205866 | Bukhansan, Seoul, Kyunggi-do |
| 12 | Brown rat | *R. norvegicus* | MuRaNo-3 | **o** | **o** | **o** | **o** | **o** | **x** | ON205878 | Geosang, Gyeongsangnam-do |
| 13 | Water dear | *H. inermis* | CeHyIn-3 | **o** | **o** | **o** | **o** | **o** | **x** | ON205887 | Goseong, Gangwon-do |
| 14 | Water dear | *H. inermis* | CeHyIn-5 | **o** | **o** | **o** | **o** | **o** | **x** | ON205888 | Goseong, Gangwon-do |
| 15 | Water dear | *H. inermis* | CeHyIn-7 | **o** | **o** | **o** | **o** | **o** | **x** | ON205929 | Samcheok, Gangwon-do |
| 16 | Water dear | *H. inermis* | CeHyIn-9 | **o** | **o** | **o** | **o** | **o** | **x** | ON205862 | Samcheok, Gangwon-do |
| 17 | Water dear | *H. inermis* | CeHyIn-11 | **o** | **o** | **o** | **o** | **o** | **x** | ON205858 | Samcheok, Gangwon-do |
| 18 | Water dear | *H. inermis* | CeHyIn-15 | **o** | **o** | **o** | **o** | **o** | **x** | ON205930 | Samcheok, Gangwon-do |
| 19 | Water dear | *H. inermis* | CeHyIn-21 | **o** | **o** | **o** | **o** | **o** | **x** | ON205889 | Samcheok, Gangwon-do |
| 20 | Water dear | *H. inermis* | CeHyIn-25 | **o** | **o** | **o** | **o** | **o** | **x** | ON205934 | Samcheok, Gangwon-do |
| 21 | Water dear | *H. inermis* | CeHyIn-27 | **o** | **o** | **o** | **o** | **o** | **x** | ON205935 | Samcheok, Gangwon-do |
| 22 | Water dear | *H. inermis* | CeHyIn-34 | **o** | **o** | **o** | **o** | **o** | **x** | ON205936 | Sokcho, Gangwon-do |
| 23 | Water dear | *H. inermis* | CeHyIn-35 | **o** | **o** | **o** | **o** | **o** | **x** | ON205939 | Sokcho, Gangwon-do |
| 24 | Water dear | *H. inermis* | CeHyIn-63 | **o** | **o** | **o** | **o** | **o** | **x** | ON205926 | Yeongwol, Gangwon-do |
| 25 | Water dear | *H. inermis* | CeHyIn-108 | **o** | **o** | **o** | **o** | **o** | **x** | ON205884 | Yeongwol, Gangwon-do |
| 26 | Water dear | *H. inermis* | CeHyIn-146 | **o** | **o** | **o** | **o** | **o** | **x** | ON205927 | Cheolwon, Gangwon-do |
| 27 | Water dear | *H. inermis* | CeHyIn-151 | **o** | **o** | **o** | **o** | **o** | **x** | ON205928 | Cheolwon, Gangwon-do |
| 28 | Water dear | *H. inermis* | CeHyIn-154 | **o** | **o** | **o** | **o** | **o** | **x** | ON205933 | Chuncheon, Gangwon-do |
| 29 | Water dear | *H. inermis* | CeHyIn-162 | **o** | **o** | **o** | **o** | **o** | **x** | ON205922 | Chuncheon, Gangwon-do |
| 30 | Water dear | *H. inermis* | CeHyIn-170 | **o** | **o** | **o** | **o** | **o** | **x** | ON205945 | Chuncheon, Gangwon-do |
| 31 | Water dear | *H. inermis* | CeHyIn-218 | **o** | **o** | **o** | **o** | **o** | **x** | ON205877 | Pyeongchang, Gangwon-do |
| 32 | Water dear | *H. inermis* | CeHyIn-240 | **o** | **o** | **o** | **o** | **o** | **x** | ON205943 | Taebaeksan, Gangwon-do |
| 33 | Water dear | *H. inermis* | CeHyIn-241 | **o** | **o** | **o** | **o** | **o** | **x** | ON205914 | Taebaeksan, Gangwon-do |
| 34 | Water dear | *H. inermis* | CeHyIn-250 | **o** | **o** | **o** | **o** | **o** | **x** | ON205904 | Bonghwa, Gyong-buk-do |
| 35 | Water dear | *H. inermis* | CeHyIn-254 | **o** | **o** | **o** | **o** | **o** | **x** | ON205915 | Bonghwa, Gyong-buk-do |
| 36 | Water dear | *H. inermis* | CeHyIn-262 | **o** | **o** | **o** | **o** | **o** | **x** | ON205908 | Pyeongchang, Gangwon-do |
| 37 | Water dear | *H. inermis* | CeHyIn-285 | **o** | **o** | **o** | **o** | **o** | **x** | ON205902 | Yeongwol, Gangwon-do |
| 38 | Water dear | *H. inermis* | CeHyIn-290 | **o** | **o** | **o** | **o** | **o** | **x** | ON205910 | Yeongcheon, Gyeongsangbuk-do |
| 39 | Water dear | *H. inermis* | CeHyIn-297 | **o** | **o** | **o** | **o** | **o** | **x** | ON205882 | Busan city |
| 40 | Water dear | *H. inermis* | CeHyIn-298 | **o** | **o** | **o** | **o** | **o** | **o** | ON205891 | Wonju, Gangwon-do |
| 41 | Roe dear | *C. capreolus* | CeCaCa-4 | **o** | **o** | **o** | **o** | **o** | **x** | ON205859 | Goseong, Gangwon-do |
| 42 | Roe dear | *C. capreolus* | CeCaCa-5 | **o** | **o** | **o** | **o** | **o** | **x** | ON205911 | Goseong, Gangwon-do |
| 43 | Roe dear | *C. capreolus* | CeCaCa-6 | **o** | **o** | **o** | **o** | **o** | **x** | ON205912 | Goseong, Gangwon-do |
| 44 | Roe dear | *C. capreolus* | CeCaCa-7 | **o** | **o** | **o** | **o** | **o** | **x** | ON205913 | Yeongwol, Gangwon-do |
| 45 | Roe dear | *C. capreolus* | CeCaCa-12 | **o** | **o** | **o** | **o** | **o** | **o** | ON205907 | Yeongwol, Gangwon-do |
| 46 | Roe dear | *C. capreolus* | CeCaCa-13 | **o** | **o** | **o** | **o** | **o** | **x** | ON205861 | Goseong, Gangwon-do |
| 47 | Roe dear | *C. capreolus* | CeCaCa-20 | **o** | **o** | **o** | **o** | **o** | **x** | ON205871 | Goseong, Gangwon-do |
| 48 | Roe dear | *C. capreolus* | CeCaCa-21 | **o** | **o** | **o** | **o** | **o** | **x** | ON205905 | Goseong, Gangwon-do |
| 49 | Roe dear | *C. capreolus* | CeCaCa-22 | **o** | **o** | **o** | **o** | **o** | **x** | ON205906 | Goseong, Gangwon-do |
| 50 | Roe dear | *C. capreolus* | CeCaCa-23 | **o** | **o** | **o** | **o** | **o** | **x** | ON205883 | Goseong, Gangwon-do |
| 51 | Roe dear | *C. capreolus* | CeCaCa-24 | **o** | **o** | **o** | **o** | **o** | **x** | ON205903 | Hwacheon, Gangwon-do |
| 52 | Wild boar | *S. scrofa* | SuSuSc-29 | **o** | **o** | **o** | **o** | **o** | **x** | ON205897 | Chuncheon, Gangwon-do |
| 53 | Wild boar | *S. scrofa* | SuSuSc-30 | **o** | **o** | **o** | **o** | **o** | **x** | ON205855 | Chuncheon, Gangwon-do |
| 54 | Wild boar | *S. scrofa* | SuSuSc-33 | **o** | **o** | **o** | **o** | **o** | **o** | ON205890 | Chuncheon, Gangwon-do |
| 55 | Leopard cat | *P. bengalensis* | CaPrBe-45 | **o** | **o** | **o** | **o** | **o** | **x** | ON205937 | Gure, Gyeongsangnam-do |
| 56 | Leopard cat | *P. bengalensis* | CaPrBe-46 | **o** | **o** | **o** | **o** | **o** | **x** | ON205921 | Gure, Gyeongsangnam-do |
| 57 | Leopard cat | *P. bengalensis* | CaPrBe-49 | **o** | **o** | **o** | **o** | **o** | **x** | ON205864 | Inje, Gangwon-do |
| 58 | Leopard cat | *P. bengalensis* | CaPrBe-54 | **o** | **o** | **o** | **o** | **o** | **x** | ON205895 | Taebaeksan, Gangwon-do |
| 59 | Leopard cat | *P. bengalensis* | CaPrBe-57 | **o** | **o** | **o** | **o** | **o** | **x** | ON205893 | Taebaeksan, Gangwon-do |
| 60 | Leopard cat | *P. bengalensis* | CaPrBe-73 | **o** | **o** | **o** | **o** | **o** | **x** | ON205918 | Bonghwa, Gyong-buk-do |
| 61 | Leopard cat | *P. bengalensis* | CaPrBe-74 | **o** | **o** | **o** | **o** | **o** | **x** | ON205920 | Bonghwa, Gyong-buk-do |
| 62 | Leopard cat | *P. bengalensis* | CaPrBe-77 | **o** | **o** | **o** | **o** | **o** | **x** | ON205899 | Yeongwol, Gangwon-do |
| 63 | Leopard cat | *P. bengalensis* | CaPrBe-80 | **o** | **o** | **o** | **o** | **o** | **x** | ON205916 | Yangyang,Gangwon-do |
| 64 | Leopard cat | *P. bengalensis* | CaPrBe-81 | **o** | **o** | **o** | **o** | **o** | **x** | ON205909 | Yangyang,Gangwon-do |
| 65 | Leopard cat | *P. bengalensis* | CaPrBe-82 | **o** | **o** | **o** | **o** | **o** | **x** | ON205857 | Yangyang,Gangwon-do |
| 66 | Leopard cat | *P. bengalensis* | CaPrBe-91 | **o** | **o** | **o** | **o** | **o** | **x** | ON205900 | Yangyang,Gangwon-do |
| 67 | Leopard cat | *P. bengalensis* | CaPrBe-97 | **o** | **o** | **o** | **o** | **o** | **x** | ON205932 | Sokcho, Gangwon-do |
| 68 | Leopard cat | *P. bengalensis* | CaPrBe-98 | **o** | **o** | **o** | **o** | **o** | **x** | ON205938 | Sokcho, Gangwon-do |
| 69 | Least weasel | *Mustella nivalis* | CaMuNi-1 | **o** | **o** | **o** | **o** | **o** | **x** | ON205863 | Taebaeksan, Gangwon-do |
| 70 | Siberian weasel | *M.siberica* | CaMuSi-9 | **o** | **o** | **o** | **o** | **o** | **x** | ON205856 | Sokcho, Gangwon-do |
| 71 | Yellow-throated marten | *M. flavigula* | CaMaFl-9 | **o** | **o** | **o** | **o** | **o** | **x** | ON205876 | Yangyang, Gangwon-do |
| 72 | Yellow-throated marten | *M. flavigula* | CaMaFl-12 | **o** | **o** | **o** | **o** | **o** | **x** | ON205901 | Yeongwol, Gangwon-do |
| 73 | Badger | *Meles meles* | CaMeMe-1 | **o** | **o** | **o** | **o** | **o** | **x** | ON205925 | Chuncheon, Gangwon-do |
| 74 | Badger | *M. meles* | CaMeMe-2 | **o** | **o** | **o** | **o** | **o** | **x** | ON205898 | Yangyang, Gangwon-do |
| 75 | Badger | *M. meles* | CaMeMe-3 | **o** | **o** | **o** | **o** | **o** | **x** | ON205853 | Taebaeksan, Gangwon-do |
| 76 | Badger | *M. meles* | CaMeMe-4 | **o** | **o** | **o** | **o** | **o** | **x** | ON205854 | Chuncheon, Gangwon-do |
| 77 | Badger | *M. meles* | CaMeMe-12 | **o** | **o** | **o** | **o** | **o** | **x** | ON205867 | Daeryongsan, Chuncheon, Gangwon-do |
| 78 | Badger | *M. meles* | CaMeMe-13 | **o** | **o** | **o** | **o** | **o** | **x** | ON205892 | Daeryongsan, Chuncheon, Gangwon-do |
| 79 | Siberian flying squirrel | *Pteromys volans* | RoPtVo-1 | **o** | **o** | **o** | **o** | **o** | **x** | ON205894 | Taebaeksan, Gangwon-do |
| 80 | Siberian flying squirrel | *P. volans* | RoPtVo-11 | **o** | **o** | **o** | **o** | **o** | **x** | ON205931 | Chuncheon, Gangwon-do |
| 81 | Magpie | *Pica serica* | ABF-17 | **o** | **o** | **o** | **o** | **o** | **x** | ON205873 | Samcheok, Gangwon-do |
| 82 | Magpie | *P. serica* | ABF-41 | **o** | **o** | **o** | **o** | **o** | **x** | ON205860 | Samcheok, Gangwon-do |
| 83 | Magpie | *P. sericea* | ABF-42 | **o** | **o** | **o** | **o** | **o** | **x** | ON205861 | Hongcheon, Gangwon-do |
| 84 | Magpie | *P. sericea* | ABF-48 | **o** | **o** | **o** | **o** | **o** | **x** | ON205874 | Chuncheon, Gangwon-do |
| 85 | Magpie | *P. sericea* | ABF-63 | **o** | **o** | **o** | **o** | **o** | **x** | ON205923 | Chuncheon, Gangwon-do |
| 86 | Magpie | *P. sericea* | ABF-64 | **o** | **o** | **o** | **o** | **o** | **x** | ON205924 | Chuncheon, Gangwon-do |
| 87 | Magpie | *P. sericea* | ABF-65 | **o** | **o** | **o** | **o** | **o** | **x** | ON205941 | Chuncheon, Gangwon-do |
| 88 | Magpie | *P. sericea* | ABF-66 | **o** | **o** | **o** | **o** | **o** | **x** | ON205940 | Chuncheon, Gangwon-do |
| 89 | Magpie | *P. sericea* | ABF-67 | **o** | **o** | **o** | **o** | **o** | **x** | ON205872 | Chuncheon, Gangwon-do |
| 90 | Magpie | *P. sericea* | ABF-68 | **o** | **o** | **o** | **o** | **o** | **x** | ON205896 | Chuncheon, Gangwon-do |
| 91 | Magpie | *P. sericea* | ABF-72 | **o** | **o** | **o** | **o** | **o** | **x** | ON205917 | Chuncheon, Gangwon-do |
| 92 | Magpie | *P. sericea* | ABF-74 | **o** | **o** | **o** | **o** | **o** | **x** | ON205944 | Chuncheon, Gangwon-do |
| 93 | Magpie | *P. sericea* | ABF-88 | **o** | **o** | **o** | **o** | **o** | **x** | ON205875 | Chuncheon, Gangwon-do |
|  | **Total** | | | **93** | **93** | **93** | **93** | **93** | **5** |  |  |

^#^EMB=*E.coli*-selective eosin methylene blue; CT-SMAC= STEC selective cefixime tellurite sorbitol MacConkey (CT-SMAC) agar media; ‘O'=detected, "X'=not detected; "$'=based on isoleucine--tRNA ligase gene sequences of *stx1*-positive *E. coli*
