# Supplementary material for: Prevalence of Salmonella spp. and Escherichia coli in the feces of free-roaming wildlife throughout South Korea
Source: PLoS One. 2024 Feb 15;19(2):e0281006. doi: 10.1371/journal.pone.0281006 (PMC10868816; doi:10.1371/journal.pone.0281006)
Supplement: S3 Table — (DOCX) [file pone.0281006.s010.docx]

**S3 Table**. Monthly and seasonal sample information and prevalence of *Salmonella*, *Escherichia coli*, Shiga toxin genes (*stx1*), and (*stx1*+*stx2*)-detected *Escherichia*

| Year | **Season** | **Month** | **No. tested fecal sample** | **Percentage of detected sample (No. positive sample)** ^***^ | | | |
| --- | --- | --- | --- | --- | --- | --- | --- |
|  |  |  |  | **No. of *Salmonella*-detected sample** | ***E. coli*** | ***stx1*-positive *E. coli*** | ***stx1*+*stx2-*positive *E. coli*** |
| 2015 | Autumn | Sep | 7 | 0 (0/7) | 42.86 (3/7) | 14.29 (1/7) | 0 (0/7) |
|  |  | Oct | 5 | 0 (0/6) | 0 (0/6) | 0 (0/6) | 0 (0/6) |
|  |  | Nov | 3 | 0 (0/3) | 50.00 (1/3) | 0 (0/3) | 0 (0/3) |
|  |  | Subtotal | 15 | 0 (0/15) | 26.7(4/15) | 6.7 (1/15) | 0 (0/15) |
| 2016 | Spring | May | 28 | 7.14 (2/28) | 60.71 (17/28) | 21.43 (5/28) | 3.57 (1/28) |
|  |  | Subtotal | 28 | 7.14 (2/28) | 60.71 (17/28) | 17.85 (6/28) | 3.57 (1/28) |
|  | Summer | Jun | 110 | 8.18 (9/110) | 55.45 (61/110) | 4.45 (6/110) | 0.91 (1/110) |
|  |  | Jul | 122 | 3.28 (4/122) | 41.80 (51/122) | 17.21 (21/122) | 0 (0/122) |
|  |  | Aug | 52 | 0 (0/52) | 25 (13/52) | 3.85 (2/52) | 0 (00/52) |
|  |  | Subtotal | 284 | 4.6 (13/284) | 44.0 (125/284) | 10.21 (29/284) | 0.35 (1/284) |
|  | Autumn | Sep^@^ | 95 | 0 (0/95) | 30.53 (29/95) | 7.37 (7/95) | 0 (0/95) |
|  |  | Oct | 23 | 13.04 (3/23) | 39.13 (9/23) ** | 39.13 (9/23) ** | 0 (0/23) |
|  |  | Nov | 15 | 6.66 (1/15) | 33.33 (5/15) ** | 0 (0/15) | 0 (0/15) |
|  |  | Subtotal | 133 | 3.0 (4/133) | 32.3 (43/133) | 12.0 (16/133) | 0 (0/133) |
| 2017 | Spring | Mar | 58 | 0 (0/58) | 46.55 (27/58) * | 3.45 (2/58) | 0 (0/58) |
|  |  | Apr | 24 | 0 (0/24) | 70.83 (17/24) ** | 25.00 (6/24) ** | 0 (0/24) |
|  |  | May | 41 | 2.44 (1/41) | 60.98 (25/41) ** | 29.27 (12/41) ** | 2.44 (1/41) |
|  |  | Subtotal | 123 | 0.81 (1/123) | 56.1 (69/123) | 16.26 (20/123) | 0.81 (1/123) |
|  | Summer | Jun | 59 | 1.69 (1/59) | 57.63 (34/59) ** | 11.86 (7/59) | 0 (0/59) |
|  |  | Jul | 47 | 0 (0/47) | 51.06 (24/47) ** | 29.79 (14/47) ** | 4.26 (2/47) |
|  |  | Aug | 10 | 0 (0/10) | 30.00 (3/10) | 0 (0/10) | 0 (0/10) |
|  |  | Subtotal | 116 | 0.86 (1/116) | 52.59 (61/116) | 18.10 (21/116) | 1.72 (2/116) |
| Total |  | Total | 699 | 3.004 (21/699) | 45.76 (319/699) | 13.304 (93/699) | 0.72 (5/699) |

^‘***’^The numbers in parentheses indicate the percentage with number of the pathogen-detected samples divide by tested samples in close bracket.

‘**’ indicates *P*<0.001 (in case of *Escherichia coli*, seven months showed the significant either *P*<0.001 or <0.005, except August, *P*<0.169; in case of STEC, four months (April, July, May, and October) showed the significant *P*<0.001); ‘*’indicates the *P*<0.005. ^“@”^ September is a baseline month compared to rest of eight months.

| Year | **Season** | **Month** | **No. tested fecal sample** | **Percentage of detected sample (No. positive sample)** ^***^ | | | |
| --- | --- | --- | --- | --- | --- | --- | --- |
|  |  |  |  | **No. of *Salmonella*-detected sample** | ***E. coli*** | **Stx1 detected *E. coli*** | **Stx1+Stx2 detected *E. coli*** |
| 2015 | Autumn | Sep | 7 | 0 (0/7) | 42.86 (3/7) | 14.29 (1/7) | 0 (0/7) |
|  |  | Oct | 5 | 0 (0/6) | 0 (0/6) | 0 (0/6) | 0 (0/6) |
|  |  | Nov | 3 | 0 (0/3) | 50.00 (1/3) | 0 (0/3) | 0 (0/3) |
|  |  | Subtotal | 15 | 0 (0/15) | 26.7(4/15) | 6.7 (1/15) | 0 (0/15) |
| 2016 | Spring | May | 28 | 7.14 (2/28) | 60.71 (17/28) | 21.43 (5/28) | 3.57 (1/28) |
|  |  | Subtotal | 28 | 7.14 (2/28) | 60.71 (17/28) | 17.85 (6/28) | 3.57 (1/28) |
|  | Summer | Jun | 110 | 8.18 (9/110) | 55.45 (61/110) | 4.45 (6/110) | 0.91 (1/110) |
|  |  | Jul | 122 | 3.28 (4/122) | 41.80 (51/122) | 17.21 (21/122) | 0 (0/122) |
|  |  | Aug | 52 | 0 (0/52) | 25 (13/52) | 3.85 (2/52) | 0 (00/52) |
|  |  | Subtotal | 284 | 4.6 (13/284) | 44.0 (125/284) | 10.21 (29/284) | 0.35 (1/284) |
|  | Autumn | Sep | 95 | 0 (0/95) | 30.53 (29/95) | 7.37 (7/95) | 0 (0/95) |
|  |  | Oct | 23 | 13.04 (3/23) | 39.13 (9/23) | 39.13 (9/23) | 0 (0/23) |
|  |  | Nov | 15 | 6.66 (1/15) | 33.33 (5/15) | 0 (0/15) | 0 (0/15) |
|  |  | Subtotal | 133 | 3.0 (4/133) | 32.3 (43/133) | 12.0 (16/133) | 0 (0/133) |
| 2017 | Spring | Mar | 58 | 0 (0/58) | 46.55 (27/58) | 3.45 (2/58) | 0 (0/58) |
|  |  | Apr | 24 | 0 (0/24) | 70.83 (17/24) | 25.00 (6/24) | 0 (0/24) |
|  |  | May | 41 | 2.44 (1/41) | 60.98 (25/41) | 29.27 (12/41) | 2.44 (1/41) |
|  |  | Subtotal | 123 | 0.81 (1/123) | 56.1 (69/123) | 16.26 (20/123) | 0.81 (1/123) |
|  | Summer | Jun | 59 | 1.69 (1/59) | 57.63 (34/59) | 11.86 (7/59) | 0 (0/59) |
|  |  | Jul | 47 | 0 (0/47) | 51.06 (24/47) | 29.79 (14/47) | 4.26 (2/47) |
|  |  | Aug | 10 | 0 (0/10) | 30.00 (3/10) | 0 (0/10) | 0 (0/10) |
|  |  | Subtotal | 116 | 0.86 (1/116) | 52.59 (61/116) | 18.10 (21/116) | 1.72 (2/116) |
| Total |  | Total | 699 | 3.004 (21/699) | 45.76 (319/699) | 13.304 (93/699) | 0.72 (5/699) |

^***^The numbers in parentheses indicate the percentage with number of the pathogen-detected samples divide by tested samples in close bracket.
